# Research protocol for a diagnostic study of non-invasive exhaled breath analysis for the prediction of oesophago-gastric cancer

**DOI:** 10.1136/bmjopen-2015-009139

**Published:** 2016-01-06

**Authors:** Sheraz R Markar, Jesper Lagergren, George B Hanna

**Affiliations:** 1 Division of Surgery, Department of Surgery and Cancer, Imperial College London, London, UK; 2 Department of Molecular Medicine and Surgery, Karolinska Institutet, Stockholm, Sweden

**Keywords:** SURGERY

## Abstract

**Introduction:**

Despite improvements in a range of chemo, radio and surgical therapies, the overall survival at 5 years from oesophago-gastric cancer remains poor and ranges from 10% to 30%. Early diagnosis is a key strategy to improve survival but early disease stage has non-specific symptoms that are very common while the warning clinical picture often indicates advanced disease. The aim of this research is to validate a breath test to predict oesophago-gastric cancer therefore allowing earlier diagnosis and introduction of treatment.

**Methods and analysis:**

The study will include 325 patients and be conducted across four major oesophago-gastric cancer centres in London, UK. This research will utilise selected ion flow-tube mass spectrometry (SIFT-MS) exhaled breath analysis, for comparison of predicted cancer risk based on the previously developed volatile organic compound exhaled breath model, with endoscopic findings and histology biopsies. This will determine the overall diagnostic accuracy for non-invasive breath testing for the diagnosis of oesophago-gastric cancer.

**Ethics and Dissemination:**

Approval was gained from NRES Committee London, on 16 July 2014 (REC reference 14/LO/1136) for the completion of this study. Different methods of dissemination will be employed including international clinical and patient group presentations, and publication of research outputs in a high-impact clinical journal. This is to ensure that the findings from this research will reach patients, primary care practitioners, scientists, hospital specialists in gastroenterology, oncology and surgery, health policymakers and commissioners as well as NHS regulatory bodies.

**Trials registration number:**

UKCRN18063; Pre-results.

## Introduction

In the UK, upper gastrointestinal (GI) symptoms account for at least 3% of consultations in primary care,^1^ and the national oesophago-gastric (OG) cancer audit (2013) suggested the number of patients per annum diagnosed with OG cancer was approximately 11 500 with only 35% treated with a curative intent.^2^ Current UK referral guidelines for OG cancer focus on alarm symptoms such as dysphagia and odynophagia, despite these symptoms having poor sensitivity and specificity for cancer, and often only occurring in advanced disease, translating into poor outcome and overall survival. There is a wide range in the rates of OG-duodenoscopy (OGD) performed among general practice populations in England, and it appears on average that patients with OG cancer belonging to practices with the lowest rates of OGD performed are at greater risk of poorer overall outcome.^3^

OGD remains the gold-standard investigation for the assessment of patients with upper GI symptoms and considered at risk of OG cancer. OGD is an expensive investigation, uncomfortable for the patient, and not without important risks including visceral perforation and bleeding. Furthermore, due to a lack of clear guidance regarding the utilisation of OGD, the identification of OG cancer currently only occurs in 2% of all OGDs in the UK,^3^ and often at a late and incurable stage.

Our group undertook a systematic review to evaluate the clinical evidence for the utilisation of volatile organic compound (VOC) analysis from breath in the assessment of GI disease.^4^ Eleven studies comprising 934 patients were included. Significant differences in the VOC profiles from exhaled breath of patients with gastro-oesophageal cancer were observed, suggesting this may have a future role as a non-invasive diagnostic test. No studies reported any adverse events associated with VOC breath analysis. Breath analysis has been shown to be acceptable and of diagnostic value in routine clinical practice for the detection of *Helicobacter Pylori*^5^ and intestinal bacterial overgrowth,^6^ and in the diagnosis and assessment of asthma.^7^ ^8^

Previous research undertaken by our group, using selected-ion flow-tube mass spectrometry (SIFT-MS), demonstrated the presence of specific VOCs from the headspace of urine and gastric content that were associated with OG cancer.^9^ ^10^ Following this, our group focused research on the analysis of VOCs from exhaled breath. In an initial pilot study, we found four VOCs that significantly differed between patients with cancer and positive-control patients.^11^ We extended this initial research to a larger cohort of 220 patients in whom we developed a model based on the analysis of 12 VOCs from exhaled breath, with a sensitivity of 84.6% and specificity of 76.1%, for the prediction of OG adenocarcinoma.^12^ Further work undertaken over the past year in a follow-up cohort of 60 patients further refined this diagnostic VOC breath model to nine VOCs (propanoic acid, butyric acid, pentanoic acid, hexanoic acid, pentanal, heptanal, octanal, nonanal and decanal) from two chemical groups (fatty acid and aldehyde) (sensitivity 95% and specificity 69%) with improved mechanistic understanding of their derangement in OG adenocarcinoma. Previous research has identified specific VOC breath signatures associated with lung, biliary tract and head and neck cancers that do not overlap with the nine VOCs that will be evaluated as part of the diagnostic model for upper GI cancer in this study.^13–16^

In preparation for this multicentre study, we investigated factors that influence the loss of target VOCs from bags to eliminate time for transport between centres as a confounding factor. Samples can be stored for up to 48 h at room temperature within GastroCHECK steel breath bags with no evidence of loss of VOCs. We further studied the variation seen in the concentration of trace VOCs within ambient air, and demonstrated that this may represent a confounding factor and must be sampled regularly when undertaking multicentre breath analysis. The next stage of this research and the subject of this current proposal is to externally validate the model for the prediction of OG cancer in a multicentre setting.

Analysis of VOCs from breath provides a non-invasive risk stratification tool to identify high-risk patients who should be referred for OGD at an earlier stage. This will provide a more rational approach to the utilisation of OGD and may allow earlier identification of less invasive and potentially curable cancer. The external validation of this VOC breath model in a large multicentre study will provide this risk-stratification tool that triages the patient to have endoscopy. The aim of this present study is to determine the diagnostic accuracy of an exhaled breath test in the prediction of OG cancer in a multicentre blind validation study.

## Methods and analysis

**Main centre(s):** St Mary's, Royal Marsden Hospital, Queens Romford and University College London Hospitals, London, UK.

### Design

- **Inclusion criteria:** Patients aged more than 18 years with upper GI symptoms attending for endoscopy or surgery. In the cancer cohort, only patients with non-metastatic OG adenocarcinoma (stage I–III) will be included.
- **Exclusion criteria:** Patients with a documented active infection or known liver failure, and those unable to provide informed consent or unable to provide a 500 mL breath sample.
- **Intervention(s) or method**
- *Breath sampling methodology:* All patients will be asked to sign a consent form to be included in this study (see online supplementary appendix A), and will be given an information leaflet (see online supplementary appendices B and C). Patients will fast for a minimum of 6 h prior to their breath sample collection. Patients will rest in the same area for at least 20 min prior to breath sampling and all breath samples will be retrieved prior to endoscopy. Patients will be asked to perform a single deep nasal inhalation followed by complete exhalation via their mouth into a secure GastroCHECK steel breath bag (500 mL) via a 1 mL Luer-Lok syringe (Terumo Europe, Leuven, Belgium). For each VOC measurement, the syringe plunger will be removed from the 1 mL Luer-Lok syringe and the GastroCHECK bag will be directly connected via the syringe barrel to the sample inlet arm of the SIFT-MS instrument. Patients with OG adenocarcinoma will be sampled prior to the induction of neoadjuvant therapy (neoadjuvant naïve).
- *VOC analysis using SIFT-MS*: The principle of SIFT-MS is as follows: selected precursor ions are formed in a microwave discharge source and are selected according to their mass-to-charge ratio, m/z, by a mass filter and injected into a helium carrier gas where they are convected as a thermalised swarm along a flow tube. H30+, NO+, O2+ precursor ions are used to ionise the trace gases in an air sample that is introduced into the helium at a known flow rate; these ions selectively ionise VOCs present within the sample, resulting in characteristic product ions. By measuring the count rate of both, precursor ions and the characteristic product ions at the downstream detection system, a real-time quantification is achieved, realising the absolute concentration of trace and volatile compounds at the parts-per-billion by volume or parts-per-million by volume. Samples will be analysed using the multi-ion monitoring mode, selective VOCs from breath will be analysed for a total of 60 s and measured concentrations will be averaged over this time for each VOC.
- **End point:** The diagnostic accuracy of the exhaled breath test for the prediction of OG cancer.

### Quality assurance

- **Calibration to water:** The concentration of water in human breath is approximately 6%. All samples will be tested using SIFT-MS to ensure that the percentage of water from the exhaled breath sample within the bag is between 5% and 6.5%. If this is not the case the sample will be discarded as it is likely to be unreliable.
- **Ambient room air:** Weekly samples will be taken from the ambient room air at the different hospitals where patients are being breath sampled and also from the laboratory air where samples are analysed. This is to ensure that there is no contamination from the ambient room air to cause anomalous results; contamination represents an important confounding factor that must be measured.
- **Standardisation of breath sampling methodology:** Human factor analysis previously undertaken by our group has shown several potential sources of error in breath sampling that can affect the results of analysis. All clinicians and researchers participating in this clinical trial will go through a credentialing process involving observation of consent, performing breath sampling and storage of samples, prior to inclusion in the study.
- **Cross-platform validation:** In a subset of 50 patients, an additional breath sample will be taken and analysed using Thermal Desorption Gas Chromatography Mass Spectrometry (TD-GC-MS). This analytical technique allows accurate compound identification, with SIFT-MS permitting accurate compound quantification.
- **Data monitoring committee:** Professor David Smith FRS (University of Keele, UK) and Professor Patrik Spanel (J Heyrovsky Institute of Physical Chemistry, Prague, Czech Republic) will provide independent data monitoring of the quality of breath analysis results obtained. Both are experts in the field of breath analysis and selected-ion flow-tube mass spectrometry (SIFT-MS). Professor Jasper Lagergren (Karolinska Institutet, Stockholm, Sweden) will monitor clinical data.

### Statistical analysis and plan, including:

- **Sample size and power calculations:** Based on 50% of patients in the study population having cancer (one benign patient will be recruited to one patient with cancer) and maintaining a sensitivity and specificity of 80% for the diagnostic model derived from our previous research, the sample size estimated for the multicentre external validation study is 325 patients; 162 patients with oesophageal or gastric cancer and 163 patients with benign conditions or a normal upper GI tract. From previous research undertaken, it is anticipated that 5% of patients recruited are likely to be withdrawn due to inconsistencies in breath sampling technique and transport. Therefore, the target recruitment for this study is 342 patients; 171 with oesophageal or gastric cancer and 171 with benign conditions or a normal upper GI tract. This sample size calculation was performed by Asif Johar, Statistician, Karolinska Institutet, Stockholm, Sweden.
- **Statistical tests:** Comparison of predicted cancer risk and actual OGD findings or histology from endoscopic biopsies (gold standard) will then be made, and the overall diagnostic accuracy (sensitivity, specificity, positive and negative predictive value, receiver operator curve analysis) for this non-invasive diagnostic investigation will be determined. Potential confounding factors across the study groups will be evaluated by employing the Kruskal-Wallis test for continuous variables and χ^2^ test for discrete variables. Linear regression models will be used to assess any influence of patient demographic factors, or medications (this data will be collected using the Case Report Form online supplementary appendix d) on VOC concentrations measured.
- **Blind analysis:** OGD findings with or without histology from endoscopic biopsies will provide the gold standard against which the results of the breath analysis model will be tested. Data will be sent to Fredrik Mattsson, senior biostatistician in Jesper Lagergren's group at Karolinska Institutet, Stockholm, Sweden, who will be blinded to the results of the OGD and histology, and he will generate the predicted cancer risk using the model previously developed and based solely on breath analysis results gained. Comparison of predicted cancer risk and actual OGD findings or histology from endoscopic biopsies will then be made.

### Ethics

- **Ethics committee approval:** NHS Health Research Authority **(**NRES Committee London—Camden and Islington) approval gained on 16 July 2014 (REC reference 14/LO/1136).
- **Interim analyses and stopping rules:** No interim analysis will be performed, and the data will analysed when the target recruitment of 325 patients has been reached.

## Dissemination

Different methods of dissemination will be employed so that the findings from this research will reach patients, primary care practitioners, scientists, hospital specialists in gastroenterology, oncology and surgery, health policymakers and commissioners as well as NHS regulatory bodies. We plan to present the findings of this research at international gastroenterology, oncology and surgical research meetings. We will also present the findings of this research to relevant patient groups including NIHR-INVOLVE and the Oesophageal Patient Association. Following generation and validation of this robust model for the prediction of OG cancer, we plan to publish the results of this research in a high impact factor clinical journal to allow widespread dissemination of this research.
